# Métis Peoples and Cancer: A Scoping Review of Literature, Programs, Policies and Educational Material in Canada

**DOI:** 10.3390/curroncol28060429

**Published:** 2021-12-04

**Authors:** Tegan Brock, Maniza Abedin Chowdhury, Tracey Carr, Adel Panahi, Marg Friesen, Gary Groot

**Affiliations:** 1 Ministry of Health, Métis Nation—Saskatchewan, Saskatoon, SK S7M 5X8, Canada; apanahi@mns.work (A.P.); margfriesen@mns.work (M.F.); 2 Department of Community Health and Epidemiology, College of Medicine, University of Saskatchewan, Saskatoon, SK S7N 5E5, Canada; mac813@mail.usask.ca (M.A.C.); tlc143@mail.usask.ca (T.C.); gary.groot@usask.ca (G.G.)

**Keywords:** Métis, cancer, scoping review, cancer screening, cancer control, cancer care, Indigenous, Canada

## Abstract

Much of the existing Indigenous cancer research focuses on First Nation populations or reports on pan-Indigenous data that include First Nations, Métis, and Inuit metrics together, which fails to capture the distinct lived realities, experiences of colonialism, and culture of each Indigenous group. The purpose of this scoping review was to summarize existing knowledge on cancer among Métis peoples in Canada, offering direction to researchers, institutions, and policymakers for future actions that enhance Métis-specific cancer surveillance and cancer care. We searched Embase, Medline, iPortal, and Proquest Theses and Dissertations databases, Google Scholar and Google, alongside ten websites relevant to cancer and Métis peoples. Two reviewers gathered 571 records. After screening, 77 records were included. Data show that Métis peoples experience higher behavioral risk factors, lower screening participation, higher cancer incidence for some cancers, and higher mortality rates compared to the non-Indigenous population. Existing research is piece-meal and researchers emphasize that there is inadequate Métis-specific cancer data. There is a need for targeted, Peoples-specific cancer control interventions to reduce these health inequities and a coordinated, Peoples-specific approach to cancer research. These efforts must involve collaboration among Métis Nations and organizations, provincial governments and agencies, researchers, and policymakers.

## 1. Introduction

There is strong evidence that current health outcomes among Indigenous populations including First Nation, Inuit and Métis (FNIM) in Canada are significantly poorer compared to those of non-Indigenous peoples in Canada [1,2,3]. (We draw from Hutchinson et al. (2018) when using different terminology referring to Indigenous peoples: “the terms First Nations, Inuit, and Métis are used to describe the three Indigenous populations in Canada. Whenever possible, First Nations and/or Inuit and/or Métis are referred to specifically, in keeping with the data source. The term Indigenous is used to describe international Indigenous peoples, with population-derived terms used where possible. The term Aboriginal remains as to reflect the original use in referenced materials” [4] (pp. 4–5). The Métis are a distinct Indigenous people and nation in the historic Northwest of Canada that emerged during the late 18th century through the fur trade. The Métis possess a culture, traditions, language (Michif), history, way of life, and nationhood unique to those of First Nations and European settlers [5]. As such, the Métis are “unique and distinct rights-bearing Aboriginal peoples and are one of three recognized Aboriginal peoples identified in subsection 35(2) of the Canadian Constitution Act, 1982 whose rights are recognized and affirmed in Section 35” [6] (p. 6). It is worth distinguishing those with mixed heritage from distinct rights-bearing Métis: “Not every person of mixed European-Aboriginal ancestry is Métis for the purposes of Section 35. Rather, it is the combination of self-identification as Métis, along with membership in larger distinct and historical Métis communities with their own unique culture, practices, traditions and languages that makes Métis distinct Aboriginal peoples and distinct from their European and other Aboriginal ancestors” [6] (p. 6).

Rates of diseases such as cardiovascular disease, diabetes, cancer, and obesity are on the rise in many Indigenous populations compared to non-Indigenous populations [7,8,9]. Cancer is a particularly concerning disease, as it has quickly risen to be one of the foremost diseases responsible for death among Indigenous populations [10,11]. In an effort to address the increasing rate of mortality due to cancer among Indigenous populations, public health scholars have turned their attention to understanding lifestyle risk factors, cancer screening rates, cancer incidence, and cancer mortality and survival rates among Indigenous populations in Canada, which are all influenced by social determinants of health [10,12,13].

While research on cancer among Indigenous peoples in Canada is growing, there remain data gaps that impede Indigenous cancer surveillance efforts [14,15,16]. Much of the existing research focuses on First Nation populations or reports on pan-Indigenous data that include First Nations, Métis, and Inuit metrics together (e.g., [17,18,19,20]). For example, a recent scoping review by Horrill et al. (2019) examined barriers to cancer care among Indigenous peoples and found 29 studies focused on First Nations populations, two that reported on First Nations and Métis populations, and three that focused on Inuit populations [13]. Despite the similarities in the health profile of these populations, there are important differences, including diverse experiences of colonization, racialization and oppression, unique cultures and traditions, and distinct histories and relationships with the government of Canada [9,21]. Studies highlight the need to consider the range of lived realities both across and within First Nations, Métis, and Inuit populations [22].

Commissioned by the National Collaborating Centre for Indigenous Health, a 2011 fact sheet explains that the absence of a true picture of population health and well-being for Métis stems from “the lack of adequate, accurate and accessible data and information on Métis health and well-being” [23] (p. 1). Young (2003) points out that measuring health data for Métis populations can be difficult because Métis peoples do not congregate in as easily discernable locations like First Nations and Inuit peoples do, with the exclusion of Métis Settlements in Alberta [24]. As a result, much of the health data available comes from national censuses or surveys, which require individuals to self-identify as Métis. Moreover, while the provision of health care for Status First Nations and Inuit lies within federal jurisdiction, health care for Métis peoples is currently the responsibility of provincial health authorities. Métis health metrics are therefore often lost within those of the general public as Canadian health databases, including cancer registries, do not use ethnocultural identifiers [15,22].

Due to the often-characterized First Nation data as ‘Aboriginal’ or ‘Indigenous’ data, it is currently unclear how much Métis cancer-specific data is available, making it difficult to summarize the degree to which cancer is impacting Métis peoples in Canada. The Canadian Partnership Against Cancer (the Partnership) has been piloting an effort to fill this gap, starting with the first Métis Cancer Control Baseline Report in 2014 which states that “data remains far short of the standard of data available for other Canadians,” and “due to these data limitations, understanding the burden and impact of cancer for Métis is a complicated task” [25] (p. 6). It is also unclear how many distinctions-based interventions, programs and educational materials targeting Métis cancer patients exist across the country.

Led by the Métis Nation-Saskatchewan Ministry of Health and in partnership with Dr. Groot and his research team at the University of Saskatchewan Department of Community Health and Epidemiology, we conducted a scoping review to identify academic and grey literature specific to knowledge about Métis cancer in Canada. Given its ability to cast a wide net, a scoping review is an appropriate tool to respond to this need by mapping existing evidence about a specific topic, by identifying the nature and extent of evidence available, and through its ability to identify parameters and gaps in available literature [26].

The purpose of this review was to summarize existing knowledge on cancer among Métis peoples in Canada, offering direction to researchers, institutions, and policymakers for future actions that enhance Métis-specific cancer surveillance and cancer care. The review had three objectives: (1) summarize the existing knowledge or data specific to Metis peoples and cancer across the cancer continuum; (2) identify gaps in available Métis-specific cancer data; and (3) summarize what jurisdictions in Canada have done for Métis populations across the cancer continuum. To address the third objective, this review also assesses the availability of Métis-specific cancer education materials, alongside cancer programs and strategies that target the Métis population.

### The Métis Nation

Métis Nation comprises the Métis National Council and its Governing Members: Métis Nation of Ontario, Métis Nation-Saskatchewan, Métis Nation of Alberta, and Métis Nation British Columbia. Mandated by the Métis National General Assembly, the Métis National Council “represents the Métis Nation at the national and international levels to advance issues of collective importance” [27]. The Governing Members’ respective Métis Legislative Assemblies are the governing bodies with the authority to enact legislation, regulations, rules, and resolutions governing the affairs and conduct of the Métis. This includes the priority of health and wellness, identified in the 2017 Crown-Métis Nation Accord [27], which outlines the collaboration between Métis Nation and the federal government on Métis-specific health research and surveillance, health policy, program development and delivery, and the “coordination, continuity and appropriateness of health services for Métis peoples” [27] (Annex 3).

## 2. Methods

We followed the framework of Arksey and O’ Malley (2005) [28] and additions in the PRISMA Extension for Scoping Reviews [29] to identify and chart relevant records.

### 2.1. Sources

Given the broad extent of our research questions, we searched a variety of search engines, databases, and websites to identify academic and grey records. With the assistance of a Health Sciences Librarian from the University of Saskatchewan, we identified four databases, two search engines, and ten websites (see Table 1) to search. Websites were chosen based on their relevance to the research objectives and to ensure we captured programs, services, strategies, and material resources; provincial sites were chosen due to their high population of Métis peoples and to align with provincial Métis governments. To find relevant grey documents, programs, services, and educational materials related to Métis and cancer in Canada, websites were hand-searched and are therefore not entirely replicable.

### 2.2. Search Strategy

Searches were conducted between April 2020–July 2020 by T.B. and M.A.C. We did not limit the dates for our searches as cancer is a relatively newly conceptualized disease among Métis populations, and our searches were conducted in English. The research objectives informed the search terms, and we applied different search terms for academic and grey literature searches (see Table 2). For example, to find Métis-specific information, academic searches focused on Métis content only (not First Nation or Inuit); academic searches also used terms for the four most common cancer types (colon, breast, cervical, and lung), and terms for the following three categories: ‘experience/support,’ ‘policy/strategy’ and ‘community support for patients.’ For academic searches, the Boolean operator “OR” was used to join keywords within the same category, and the Boolean operator “AND” was used to join all major components. Grey sources were searched using ‘Métis’ as well as ‘Indigenous’ or ‘Aboriginal’ because many programs, supports, and educational materials target that population as a whole; we also searched ‘cancer’ broadly (see Table 2). To capture theses and dissertations we searched ProQuest Dissertations and Theses, limiting our search to Canada. Given the high yield of results, we limited our screening to the first 30 hits, at which point all of them were either duplicates or not relevant to our study. Google Scholar and Google were screened until results yielded only duplicate sources or no relevant sources, ranging between 30–50 results.

We also scanned the references of included publications to identify further records. There was one hand-selected record known through our partnership with the University of Saskatchewan that we searched for additional records [30]. Both reviewers carried out the same searches, recognizing that website searches are not exactly the same. Records were tracked using Zotero reference managing software and once searches were complete, they were transferred to Covidence [31] for screening and data extraction.

### 2.3. Record Selection Process

We selected records in two phases. First, two independent reviewers (M.A.C. & T.B.) screened titles, abstracts, and introductory paragraphs of all records (*n* = 402) using the inclusion and exclusion criteria presented in Table 3. Reviewers discussed any conflicts and came to a consensus to include or exclude records for the next round of screening. Next, the same reviewers each completed a full-text review of the remaining 165 records. Reviewers paid particular attention to the methods used to determine if the data source was representative of Métis peoples and ensure that information related to both cancer and Métis peoples.

**Figure 1:**
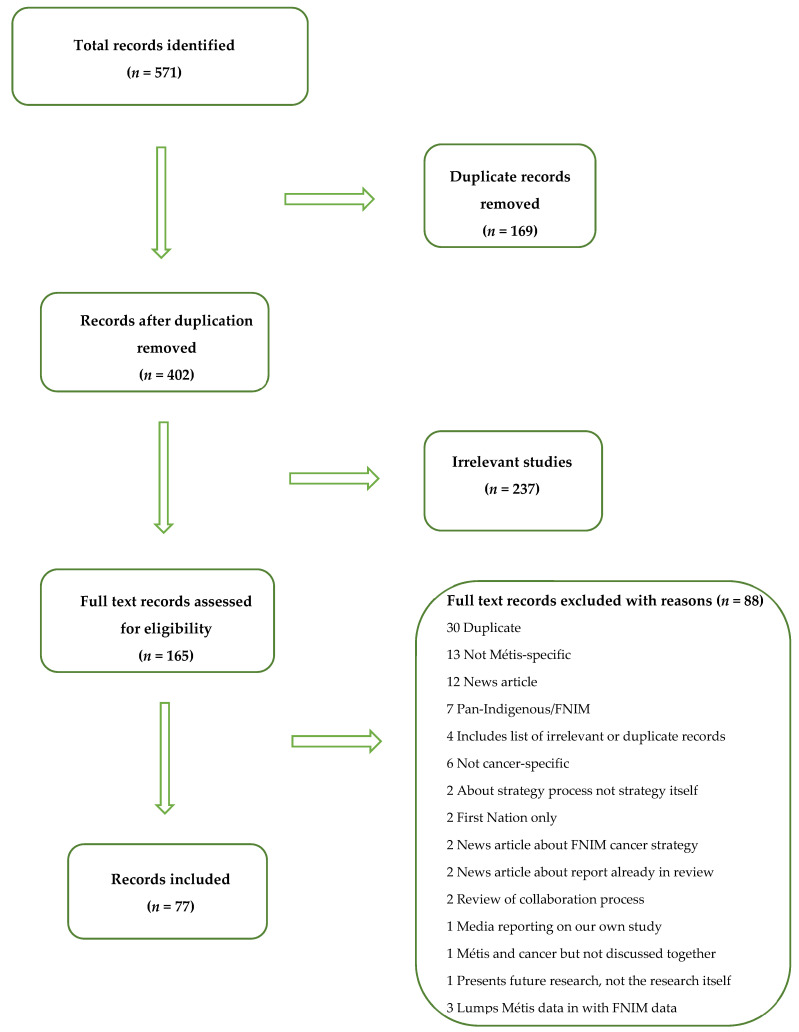
Summary of the screening and selection process.

Records were excluded if they discussed Métis populations and cancer separately or if they addressed cancer in Indigenous/First Nation, Inuit, and Métis populations, but the data from each group were lumped together (i.e., reporting findings under the ‘Aboriginal/Métis’ or ‘Indigenous’ category); news articles were also excluded. While ‘future research’ was not on our original exclusion list, we decided to exclude records if they solely proposed future research because the data was not yet available. For records without an abstract, full text copies were collected to screen. Thirteen disputes between the two reviewers were resolved by dialogue, and a final decision was made collaboratively. Figure 1 outlines the screening and selection process; seventy-seven records were included in the review.

### 2.4. Data Extraction

We used a data extraction template to track and gather relevant information from final records. The template was built in Covidence (see Appendix A) and accommodated a range of content (e.g., academic articles, reports, cancer control plans, programs/services, and educational material). We did not assess the quality of research as that analysis sits outside the purview of scoping reviews and was not relevant to our study’s purpose. For research-oriented records, the template was tailored to capture data items such as jurisdiction, research type, methods, data source, research questions or objectives, and key research findings relevant to Métis and cancer. For non-research-related records, the template captured data items such as jurisdiction, target audience, key components of program/service/strategy, and a key focus on educational materials. After the reviewers tested the template with a few records, it was adapted to better organize data items.

Data extraction was done by two reviewers, with each reviewer extracting half of the records. During this process, the two reviewers discussed and solved any uncertainty about relevant information from records.

### 2.5. Data Charting

To interpret and analyze the extracted data, information from the Covidence templates was transferred to Microsoft Excel sheets and further summarized in tables using Microsoft Word. Information was first categorized by record type (e.g., academic, grey, program/service, policy/plan, educational materials) into Excel sheets, and analyzed categorically by TB. Through this initial analysis, categories for cancer focus areas, key findings, and research gaps were identified, refined, and summarized as themes into Microsoft Word tables. Tables summarizing grey records, programs, plans, and educational materials were organized by jurisdiction and included service type and target audience information as well; all tables included analysis observations and notes. Key findings were reviewed together to identify similarities and differences between them and to generate themes such as data access/availability, screening outcomes and cancer incidence. This allowed for the identification of relationships and comparison between themes and record types, and assisted in the reporting of results.

## 3. Summary of Evidence

A total of 77 records were included in the review (13 peer-reviewed, 21 grey, 24 educational materials, 10 programs/services, and 9 plans/strategies). While the latter three categories fall into the ‘grey literature’ bucket, we chose to review them separately to ensure the findings addressed the different audiences identified in our purpose statements (researchers, institutions, and policymakers). Results are presented according to record type.

### 3.1. Academic Literature

We identified 13 peer-reviewed records related to Métis and cancer. Table 4 summarizes the datasets used by academic quantitative studies as well as the jurisdictions covered, method type (*n* = 4 qualitative and *n* = 9 quantitative studies), focus area, and key findings. Eight studies were published within the last five years, and the remaining five were published within the last ten years, indicating that academic Métis-specific cancer research may be a more recent occurrence. Most of the data are older, ranging from 1991–2011, and the Canadian Censuses, Canadian Community Health Survey, and Aboriginal Peoples Survey all used self-identification as indicators for Métis ethnicity; only one study linked to Métis registry data [32]. Most of these studies required the linking of multiple datasets to access Métis-specific health and cancer information. Four studies collaborated with an Indigenous Nation, community, or organization [32,33,34,35], and of the remaining nine, six involved statistical analyses using survey data.

Three out of the thirteen academic studies focused solely on Métis populations [32,38,39], four included FNIM data with each Indigenous groups’ data reported separately [4,37,40,42], and three included data for First Nations and Métis with each group reported on separately [33,41,43]. Three studies focused broadly on FNIM as they studied services and programs geared towards Indigenous populations [34,35,36]. The type of cancer-related focus was relatively balanced across academic studies, with some papers covering more than one focus area. Six studies considered sociodemographic variables in their analyses including ethnic identity, education, household income [33]; family physician [38]; age, gender, marital status, education, location of residence, and geographic remoteness [40]; income, education, employment and housing [42]; education, sex, age, income, residence in urban/rural location [41]; and income and rurality [43]. Two records acknowledged colonization [43] and institutional racism [42] as determinants of health among First Nations and Métis [43]; three acknowledged colonization [36], assimilation [37] and systemic racism [34] as factors that impact FNIM health; two noted that impacts of colonization [4,35] and racism [4] result in barriers to cancer care or inequitable and culturally inadequate care; and six records did not mention colonization or systemic racism at all.

Almost all academic records reported a lack of Métis-specific cancer data and how this limits cancer surveillance for Métis populations. Additionally, Sanchez-Ramirez et al. (2016) and Withrow et al. (2014) emphasized the need for consistent data on Métis-specific survival and mortality rates as well as cancer incidence and behavioral risk factors for Métis cancer surveillance and control measures to be effective [32,42].

### 3.2. Grey Literature

We identified twenty-one records from the grey literature related to Métis peoples and cancer, summarized in Table 5. There were twelve qualitative and nine quantitative studies, and the majority were published within the last ten years (16), five of which were published between 2016–2020. Just under half (9) focused on First Nation, Métis, and Inuit populations together, while the other twelve focused solely on Métis populations, including at the national level (2), provincial level (Manitoba = 4, Ontario = 3, Alberta = 2), and specific to Métis women (1). While grey literature did not directly consider sociodemographic variables, four records mentioned social determinants of health as important context for cancer data and control [8,23,44,45], nine records identified discrimination, racism or colonization as a factor that influences health status and inequities [25,30,44,46,47,48,49,50,51], and eight records did not mention any of these considerations [21,32,52,53,54,55,56,57]. Furthermore, Métis Centre of the National Aboriginal Health Organization (2011) and Métis Nation of Ontario and Cancer Care Ontario (2015) highlight that there is a need for research that assesses sociodemographic factors among Métis populations alongside Métis health data [23,49].

The Partnership published five out of the fourteen reports; other records were led and authored by provincial Métis governments, provincial cancer or health agencies, and the National Collaborating Centre for Indigenous Health; one report was led by researchers from the University of Saskatchewan. Sixteen projects involved Indigenous leadership and collaboration.

Grey literature focused on a range of topics across the cancer continuum. In addition to these, nine records highlighted information gaps [21,23,25,30,48,49,51,52,55], and six identified the need for culturally relevant cancer care services [25,30,46,48,51,57]. Most records demonstrated and emphasized the importance of collaboration between Indigenous nations, organizations, health research institutions, and health agencies to address information gaps and develop Métis-specific cancer surveillance programs. The benefits of such collaboration are evident in the shared authorship of eight reports, which enabled the linkage of Métis citizen registries with cancer registries, statistical data, or other health databases to identify Métis-specific cancer data, as well as enable analysis of the data.

Four records explicitly mentioned the need to take a Peoples-specific approach to cancer control, recognizing that while there may be similarities in the cancer experience among Métis, First Nations, and Inuit patients, there are also important differences that should inform prevention and care efforts [25,46,48,49].

Additionally, most records acknowledged that Métis people face barriers to cancer care and that service gaps in cancer care negatively impact Métis cancer patients. Examples of common barriers include discrimination, lack of a family physician, geographic isolation from services, and financial barriers to accessing care. Eight records explicitly stated the need for culturally relevant cancer care services for Métis patients [25,30,46,48,49,50,52,58], and four emphasized the critical role that patient navigators can play in improving cancer care for FNIM patients [30,50,51,58]. All but one mortality study indicated that cancer is a leading cause of death among Métis populations, and there was a common finding of high rates of behavioral risk factors for cancer such as smoking, alcohol consumption and lack of physical activity among Métis populations—findings that align with those presented in academic records. Reports on cancer control whose purpose was to inform national or provincial FNIM cancer control plans focus on equity, Peoples-specific approaches to prevention and cancer services, as well as the need for Peoples-specific data to generate meaningful surveillance metrics [25,46,47,48].

Overall, there was a strong message throughout the grey literature that a significant lack of Métis-specific data, including cancer data, is a major barrier to developing effective cancer control strategies. Thirteen records emphasized this data gap, with some indicating that the quality of available data is mediocre given inconsistencies across jurisdictions [23,25,52]. Fortunately, this gap was identified in reports informing cancer control plans, which recommended support for Peoples-specific data collection and surveillance.

### 3.3. Material Records

We identified twenty-five educational material records related to Métis or FNIM cancer patients, all of which involve collaboration with an Indigenous entity (Nation, organization, etc.). Table 6 summarizes the jurisdictions these records cover and their subject area, with an overwhelming majority of them created or supported by Cancer Care Ontario. There is a range of material types, including factsheets (8), flashcards (6), video links (3), patient guides (2), toolkits (2), reference manual (1), brochure (1), URL with online information (1) and comic book (1). The target audience for these records was mostly Métis peoples (19), while six targeted a broader FNIM audience. Out of those six material records, four include solely First Nation input, stories, or data despite being presented as FNIM resources.

### 3.4. Programs and Service Records

We identified ten programs and services (see Table 7), seven are in Ontario with the remaining three in Manitoba, Alberta, and B.C. While some provinces have general health service programs specific to FNIM, we only included those specific to FNIM cancer patients. Programs and services largely centered on building relationships with local Indigenous partners, patient navigation services, and cultural supports to ensure culturally appropriate care. Patient navigation services is a broad term that generally includes support for patients in understanding what to expect, how to navigate the care system, language interpretation, accessing cultural support, as well as educating care providers about Indigenous cultures. The five Indigenous Navigator services included in this review are part of Cancer Care Ontario’s Indigenous Navigator program.

### 3.5. Plans and Strategies

We identified eight cancer plans and strategies in Canada that addressed different aspects of cancer care for Métis patients from diagnosis to end-of-life care. The majority of these are from Ontario (4), followed by two national plans, one B.C. plan, and one Alberta plan. The Ontario records include two provincial FNIM strategies (Cancer Care Ontario Aboriginal Cancer Strategy II, 2011 [59] and Cancer Care Ontario’s First Nations, Inuit, Metis, and Urban Indigenous Cancer Strategy 2019–2023 [60]), and two regional cancer strategies (2015–19 Southeast Aboriginal Cancer Strategy [61] and 2015–2019 Northeast Aboriginal Cancer Plan [62]). There are also two other iterations of the Cancer Care Ontario’s Aboriginal Cancer Plan, as well as two other regional Aboriginal cancer plans (Northwest and Southwest) that our search did not pick up. The regional plans flow from priorities and objectives laid out in Ontario’s provincial cancer plans.

The Partnership authored both national records, the first in 2011, First Nations, Inuit, and Metis Action Plan on Cancer Control [63], and the 2019–2029 Canadian Strategy for Cancer Control [64], which includes First Nations, Inuit, and Métis cancer-care priorities. The 2017 B.C. cancer strategy (Improving Indigenous Cancer Journeys in BC: A Road Map) [65]) is a partnership between the First Nations Health Authority, B.C. Métis Nation, B.C. Association of Aboriginal Friendship Centers and B.C. Cancer. Lastly, the Alberta Plan is provincial in scope and not specific to FNIM peoples, however, it does recognize that Indigenous peoples are an underserved population who require ‘particular attention’ [66]. There are no Indigenous-specific cancer plans in Manitoba, Saskatchewan and Alberta, though a 28 April 2021 email from K. Marsden, Community Integration and Patient Education Specialist with Cancer Care Alberta, confirmed that Alberta is developing one.

All but one of these records are Indigenous-specific cancer control strategies that aim to address the high cancer incidence and low survival rates among Indigenous populations compared to rates in the general population. The key priorities identified across these strategies are generally similar:

- Build productive relationship-building through partnerships and collaboration (Indigenous and non-Indigenous)
- Increase screening participation rates among Indigenous populations
- Increase Indigenous cancer research and surveillance
- Prevention (including the use of culturally appropriate educational materials/outreach)
- Education (for Indigenous peoples as well as health care professionals)
- Health promotion (that is culturally relevant)
- Culturally safe and culturally appropriate cancer-related care (including programs and services)
- Palliative care (also culturally relevant)

Underlying these priorities is the goal of health equity through more appropriate and accessible cancer care for Indigenous peoples. The distinction-based FNIM Priorities laid out in the 2019–2029 Canadian Strategy for Cancer Control [64] reflect the list above, however, they differ by explicitly centering on Indigenous agency and taking a ‘Peoples-specific’ approach to cancer control, which is absent or implicit in the other strategies. This means there are Peoples-specific actions that flow from the strategy’s priorities to address cancer control for First Nations, Inuit, and Métis populations separately. The priorities are: (1) Culturally appropriate care closer to home; (2) Peoples-specific, self-determined cancer care; (3) First Nations-, Inuit-, or Métis-governed research and data systems [64].

## 4. Discussion

Results indicate that over the last decade there has been a steady focus on understanding cancer among Métis populations and cancer control for FNIM in Canada. Out of thirty-four peer-review and grey records combined, twenty-nine were published in 2011 or later, fourteen published between 2011–2014, and fifteen published between 2015–2020. Together, the academic and grey literature present a relatively balanced overview of the cancer continuum for Métis populations in Canada. While the number of quantitative and qualitative studies were even across the thirty-four academic and grey records, only one study employed interviews and focus groups with Métis cancer patients [50], though the 2009 and 2014 CPAC reports include input from Métis patients on committees and external research projects [25,46]. This demonstrates a need for more published qualitative in-depth information about Métis experiences with cancer.

There are more grey records (21) than academic records (13), indicating that Métis-specific cancer research is not necessarily entering the peer-review process. This likely stems from the purpose of Indigenous-led, health-based research, which is often meant to inform practice and policy change, rather than inform academic discussion. Researchers should therefore pay attention to lay reports when engaging in Métis health research. Also, numerous authors of academic and grey literature are involved in multiple studies, indicating that to date, Métis-specific cancer research comprises a relatively small group of researchers. Six out of thirteen academic records considered sociodemographic variables in their analyses and only one grey record summarized socioeconomic status statistics for Métis women, though thirteen grey records acknowledged the importance of considering social determinants of health on Métis health status. Only three records explicitly name colonization and/or systemic racism as determinants of health for Métis [42,43,46], though seven acknowledged that colonization and/or racism are factors that impact FNIM health [34,36,37,47,49,59,60]. Understanding how social determinants of health among Métis, including colonization and systemic racism, influence cancer experiences and outcomes is integral to addressing health inequities and should be integrated into future research [23].

All grey records were either led by an Indigenous Nation or organization or involved their collaboration, while four out of thirteen academic records mentioned Indigenous involvement. Assessing the degree of involvement of Indigenous Nations, organizations and communities is relevant because it indicates whether research is serving the priorities and needs of Indigenous communities, governments, and organizations [67].

This summary addresses our first objective to identify existing Métis-specific cancer data across the cancer continuum. Themes across the academic and grey literature were very similar, citing cancer incidence rates among Métis populations that were similar to non-Indigenous populations or in some cases higher (e.g., for bronchus, lung, female breast, liver, gallbladder, and cervical cancer); overall higher rates of behavioral risk factors than the non-Indigenous population, especially smoking and alcohol consumption; screening participation rates ranging from similar to lower compared to non-Indigenous populations; and overall higher mortality rates from cancers compared to non-Indigenous populations.

Another major theme from the literature is the paucity of Métis-specific cancer statistical data (a finding that was also the impetus for this review), with some authors offering suggestions for how to address this gap. Reasons for inadequate Métis cancer data include: cancer registries do not track ethnocultural data [25,32,37,39,52]; if registries did track cultural identity, some Métis people may be reluctant to identify due to experiences of racism and discrimination [25]; under-enumeration of Métis in provincial Métis registries which results in small sample sizes and challenges conducting statistical analyses [21,23,56]; difficulties comparing cancer control data across cancer registries due to inconsistencies in types of data sources [25,52]; multiple self-identification options in Canadian census iterations [39]; and inadequate funding of Métis-specific academic research [23].

To overcome these data limitations, most studies call for the use of Métis identifiers in provincial health and cancer registries as well as the development of comprehensive health information systems that include ethnocultural identifiers. A report on risk factors and screening behaviors for Métis in Ontario recommends the use of Métis identifiers alongside adequate and consistent Métis samples in Indigenous health research [49] (p. 8). In their review of fifty ethnocultural identification systems in Canada, Cats et al. (2012) point out that it is often a lack of political willpower and insufficient commitment to resources that hinders ethnocultural patient identification efforts [52]. Importantly, due to past and present issues of unethical research conducted ‘on’ Indigenous peoples, it is critical that any development and use of Métis patient identifiers be led by appropriate Indigenous governments and organizations.

Multiple records demonstrated the success of linking existing datasets to generate Métis cancer information, which is effective with strong partnership and collaboration. For example, through a data governance and partnership agreement, Métis Nation of Ontario (MNO) works with ICES, Ontario’s health informatics organization, to identify and analyze Ontario-Métis-specific administrative health data. This enables consistency and reliability in data to inform and support MNO’s efforts to improve the health and well-being of Métis citizens [68].

This step could be circumvented in some cases if cultural identity was tracked at the outset—a process that would require collaborative efforts to ensure cultural safety within the cancer care system. Regardless of the process, authors highlight the crucial role that long-term partnership and collaboration play in achieving appropriate data collection, data use, and data governance of Indigenous health information. Researchers call for partnership and knowledge-sharing among Indigenous governments, researchers, and data custodians [32,37,52].

Alongside highlighting data gaps identified in the literature, this review shows that much of the cancer research among Métis populations exists at the local, regional, or provincial level, and has occurred in an ad hoc manner. There are a few studies that consider Métis cancer incidence and other cancer-related information at the national level, such as Kumar & Janz (2013) [38], which used data from the 2006 Aboriginal Peoples Survey, and others [39,41,43], who used Canadian census data that are all from 2011 and earlier. This ad hoc approach is likely a consequence of the need to link provincial Métis Citizen Registries with provincial cancer registries or other databases to generate Métis-specific cancer data. While regional and provincial studies remain integral to informing local cancer control measures, the result is an incomplete national picture of cancer among Métis.

There could be a more coordinated approach to track Métis cancer data at a national level, which would provide a more comprehensive picture of how cancer is impacting Métis citizens, families, and communities, as well as provide valuable information to inform interventions and support services. The Canadian Indigenous Research Network Against Cancer may be an avenue to facilitate this work with a national scope [16]; the Partnership is also well-positioned to be involved in the national coordination of Métis-specific cancer control strategies.

This review shows that there is a trajectory towards Peoples-specific cancer control in Canada, demonstrated by literature published by provincial Métis governments, by the current national cancer strategy [64], as well as by educational materials such as Cancer Care Ontario’s Métis-specific educational brochures and fact sheets and BC’s People-specific cancer patient guidebooks. Equally important is a Peoples-specific approach to data collection and reporting, which remains less evident in the literature but is emphasized by studies included in this review [6,23,48,49,57]. To effectively inform a Peoples-specific approach to cancer control for FNIM, researchers must be more precise in their reporting on Indigenous populations by specifying when data are tied to individual Indigenous groups and avoiding pan-Indigenous claims when data are predominantly from one group.

### Strengths and Limitations

This review was robust in its methods for a few reasons. The search strategy was developed in collaboration with a university librarian specializing in health research and with experience in Indigenous health research. Two reviewers completed the searches, screening, and data extraction and consulted with one another throughout each stage to discuss and resolve any disagreements. As with any literature review, there remain some limitations: our search strategy did not include specific terminology about behavioral risk factors known to cause cancer (e.g., tobacco use, tobacco cessation) nor cancer screening terminology. We were also not able to capture comparisons of cancer data between Métis, First Nations, and Inuit, largely because the data sources were not always consistent across included records. By choosing to exclude studies that contained Métis input or data because findings reported on them together with First Nations input and data, we did not fully capture all Métis cancer research. For example, one study gathered experiences of breast cancer among Cree and Cree-Métis women in Alberta and grouped the results of all participant experiences into common themes [69]. While the findings from such a study are useful and relevant to understanding the Métis cancer experience, we decided to exclude it based on our Métis-specific criteria. This highlights the nuances of cultural identities and personal ties to kinship and raises considerations about the role of Peoples-specific research when identity is not necessarily clear-cut for participants.

## 5. Conclusions

We present a snapshot of research to date focused on understanding cancer’s impact on the Métis population in Canada, a summary of available programs, services, educational materials, and plans targeting Métis cancer patients, and recommendations to address information gaps (Table 8). We have identified a need for more focused Métis-specific cancer-related research at a larger scale, for research to consider colonization and systemic racism as social determinants of Indigenous health, and for Indigenous cancer research results to be Peoples-specific. The literature conveys that the future direction for cancer control is Peoples-specific prevention and care led by Indigenous nations, including Métis nations, and the creation of culturally relevant and safe cancer interventions, care, supports, and educational materials. These outcomes can be achieved through long-lasting and respectful partnerships between Indigenous and non-Indigenous governments, researchers, organizations, and agencies, which must continue to be built and strengthened into the future.

## Figures and Tables

**Table 1:** Academic and grey literature sources searched.

Academic Search	Grey Search
MedLine Embase iPortal (University of Saskatchewan’ s Indigenous Studies Portal) Proquest Theses & Dissertations (first 30 results) Google Scholar (first 50 results)	Google (first 50 results) Canadian Partnership Against Cancer website National Collaborating Centre for Aboriginal Health website Cancer Care Ontario website Cancer Care Alberta website Cancer Care Manitoba website BC Cancer website Métis Nation of Alberta website Métis Nation of Ontario website Manitoba Métis Federation website Métis Nation of British Colombia website

*Note.* Usask iPortal searched Cancer AND Métis to accommodate for its internal search parameters.

**Table 2:** Search terms and combinations used.

Academic Searches	Grey Searches
(Cancer OR breast cancer OR colon cancer OR cervical cancer OR lung cancer) AND (Métis OR Michif) AND (care OR experience OR support) AND Canada	We used website search functions to search for ‘Métis/Aboriginal/Indigenous’ OR ‘cancer’ depending on the nature of the website. We explored Indigenous/Aboriginal/First Nations, Inuit, and Métis tabs when available on cancer agency websites.
(Cancer OR breast cancer OR colon cancer OR cervical cancer OR lung cancer) AND (Métis OR Michif) AND (policy OR strategy) AND Canada	
(Cancer OR breast cancer OR colon cancer OR cervical cancer OR lung cancer) AND (Métis OR Michif) AND community support AND patients AND Canada	

**Table 3:** Inclusion/exclusion criteria.

Academic Records		Grey Records	
Inclusion Criteria	Exclusion Criteria	Inclusion Criteria	Exclusion Criteria
Discuss Métis and cancer together	Pan-Indigenous/FNIM approach to data and analysis (i.e., cite Indigenous when data is from First Nation studies, or lumping First Nation, Inuit or Métis data together)	Target Métis cancer patients, survivors or caregivers/family	Addresses cancer in the general public
		Target First Nation, Inuit and Métis people experiencing cancer	
English or French language	Other	English language	Other
Canada	Other	Canada	Other

**Table 4:** Jurisdiction covered, datasets used, method type, focus area and key findings of academic records.

Authors	Jurisdiction	Datasets	Qual	Quant	Focus Area	Key Findings
Abdul-Fatah, 2019 [34]	Ottawa, Ontario	NA	X		Patient navigation	Nurse Navigators in Ottawa use a patient-centered, land-based, culturally appropriate approach to provide culturally safe health care to Indigenous cancer patients. Indigenous-specific cancer care services are an effective method to reduce care inequities.
Beben & Muirhead, 2016 [36]	Canada	NA	X		Cancer control	Cancer care needs to address distinct needs of FNIM as separate groups of Indigenous peoples, rather than a pan-Indigenous approach. There needs to be more data collected that is specific to each group (FNIM) and urban/rural communities.
Cawley et al., 2018 [37]	Ontario	Canadian Community Health Survey 2007–2014		X	Behavioral risk factors	Prevalence of smoking is higher in Métis adults and adolescents than non-Indigenous adults and adolescents. Métis adolescents are more likely to be exposed to second-hand smoke than their non-Indigenous peers.
Crouse et al., 2014 [33]	Calgary, Alberta	Alberta Cancer Registry Alberta Laboratory Information System Canadian Census (1991–2011) Mortality Cohort		X	Screening rates & sociodemographic variables	Métis population in Calgary, Alberta is less likely to be screened for colorectal cancer than other ethnic groups. Having a family doctor led to higher screening participation. Sociodemographic variables seem to play a role in screening participation.
Hutchinson et al., 2018 [6]	Canada	NA	X		Screening barriers & facilitators	Screening facilitators include using culturally relevant educational materials, integrating screening opportunities with other FNIM health services, positive relationships with care providers, access to female health providers and communicating in plain language.
Kumar & Janz, 2013 [38]	Canada	Aboriginal Peoples Survey 2006		X	Screening rates & sociodemographic variable	Having a family doctor led to higher screening participation.
Mazereeuw et al., 2018 [39]	Canada	Canadian Census (1991–2011) Health and Environment Cohort; Canadian Mortality Database; Canadian Cancer Registry		X	Cancer incidence & survival	Cancer incidence among Métis populations and non-Indigenous adults is similar for many cancers. Higher incidence rates among Métis adults for female breast, liver, lung, gallbladder and cervical cancers compared to non-Indigenous adults.
McDonald & Trenholm, 2010 [40]	Northern Canada	Aboriginal Peoples Survey 2001 & 2006 Canadian Community Health Survey 2000–2002 & 2004–2005		X	Behavioral risk factors & sociodemographic variables	Prevalence of smoking is higher in Métis adults and adolescents than non-Indigenous adults and adolescents. Métis people in northern regions are twice as likely to be obese than non-Indigenous people. Métis are not significantly more likely to have binged on alcohol than non-Indigenous people. Sociodemographic variables impact behavioural risk factors and the likelihood of visiting a physician.
Sanchez-Ramirez, et al., 2016 [32]	Alberta	Métis Nation Alberta Information Registry		X	Cancer incidence & mortality	Cancer incidence among Métis populations and non-Indigenous adults is similar for many cancers. Incidence of bronchus/lung cancer was higher among Métis men than non-Métis men. No differences in cancer mortality between Métis and non-Métis people.
Sheppard et al., 2019 [35]	Ontario	NA	X		Patient navigation	Ontario Indigenous Navigator role includes symptom assessment and management, facilitating traditional healing, providing health literacy, alleviating anxiety, ensuring access to supports at the right time, and engaging with other relevant programs.
Tjepkema et al., 2011 [41]	Canada	Canadian Census (1991–2011); Canadian Mortality Database		X	Cancer mortality & sociodemographic variables	Potential years of life lost among Métis were twice those of non-Indigenous people. Cancer was a significant contributor to potential years of life lost for Métis. Sociodemographic factors are an important contributor to premature mortality rates of Métis.
Withrow et al., 2014 [42]	Ontario	Canadian Community Health Survey 2007–2011		X	Behavioral risk factors, screening rates & sociodemographic variables	Métis women are 2.5 times more likely to smoke than non-Indigenous women. Prevalence of smoking is higher in Métis adults and adolescents than non-Indigenous adults and adolescents. Métis people are twice as likely to be obese than non-Indigenous people. No significant differences in screening rates between Métis and non-Métis in Canada. (Screening rates among women are below national goals, regardless of ethnic identity (Withrow et al., 2014), so similar screening rates between Métis and non-Métis women does not necessarily indicate high or effective screening participation [42].) Ethnicity is a determinant of health-related lifestyle factors for Métis peoples.
Withrow, 2016 [43]	Canada	1991 Canadian Census Mortality Study; Canadian Cancer Registry		X	Cancer survival & sociodemographic variables	Métis have poorer survival rates for almost all of the most common cancers compared to the non-Indigenous population. Disparity remains when income and rurality are considered.

**Table 5:** Jurisdiction covered, record type, method used, and focus area of grey literature.

Author(s)	Record Type	Jurisdiction	Qualitative	Quantitative	Focus Area
Baidoobonso, 2017 [44]	Report to inform strategy/policy	Ontario	X		Screening for FNIM in Ontario
Bartlett et al., 2011 [45]	Report	Manitoba		X	Cancer incidence among Métis
Canadian Partnership Against Cancer, 2009 [46]	Report	Canada	X		FNIM cancer control
Canadian Partnership Against Cancer, 2014 [25]	Report/Document review	Canada	X		Cancer control for Métis, including behavioural risk factors and mortality
Cancer Care Ontario, 2016 [47]	Report to inform policy/strategy	Ontario	X		FNIM cancer control
Cancer Control Alberta, 2016 [58]	Report	Alberta	X		FNIM cancer care
Carter et al., 2013 [50]	Discussion paper	Manitoba	X		Métis cancer experience
Cats et al., 2012 [52]	Environmental scan	Canada	X		FNIM patient identification for cancer control; data gaps
Haver, 2014 [51]	Literature review	North America	X		FNIM patient navigator
Institute for Clinical Evaluative Sciences and Métis Nation Ontario, 2012 [55]	Lay report	Ontario		X	Lung cancer rates for Métis
Klein-Geltink et al., 2012 [56]	Lay Report	Ontario		X	Cancer incidence among Métis
Kliewer, Mayer & Wajda, 2002 [54]	Report	Manitoba		X	Cancer incidence among Métis
Martens et al., 2010 [8]	Report	Manitoba		X	Cancer incidence, screening rates and mortality among Métis
Métis Centre of the National Aboriginal Health Organization, 2012 [57]	Report	Canada		X	Cancer incidence & mortality among Métis women
Métis Centre of the National Aboriginal Health Organization, 2011 [23]	Report	Canada	X		Métis cancer data/governance
Métis Nation of Ontario and Cancer Care Ontario, 2015 [49]	Report	Ontario		X	Behavioural risk factors & screening behaviour among Métis
O’Connor, 2019 [48]	Document review	Canada	X		Cancer control for FNIM
Randall et al., 2012 [21]	Report	Alberta		X	Cancer mortality among Métis
Sanchez-Ramirez et al., 2016 [32]	Report	Alberta		X	Cancer incidence among Métis
Snelling, 2017 [53]	Report	Canada	X		FNIM cancer prevention initiatives
Groot, 2020 [30]	Environmental scan	Canada	X		FNIM cancer supports

**Table 6:** Cancer educational materials for FNIM audiences.

Jurisdiction	Title	Resource Type	Creator(s)	Subject
Alberta	Guide to Cancer Care in Alberta for Newly Diagnosed Indigenous People	Patient information guide	Alberta Health Services Cancer Control Alberta	Cancer journey/experience
Alberta	Indigenous Cancer Care Experiences: Video Series	Video series	Cancer Control Alberta Alberta Health Services Saint Elizabeth Research Centre	Survivor story Grief Traditional healing Ceremony
British Colombia	Living with Cancer—everyone deserves support (Metis version)	Patient information guide	Metis Nation BC First Nation Health Authority BC Cancer Agency BC Association of Friendship Centres	Cancer journey/experience
Ontario & British Colombia	Silent Enemy	Comic book	Cancer Care Ontario First Nations Health Authority	Story: Indigenous family dealing with cancer
National	LivingMyCulture.ca	Video series	Canadian Partnership Against Cancer Virtual Hospice	Cancer journey/experience
Ontario	Be Tobacco-Wise—Métis	Brochure	Cancer Care Ontario	Prevention Tobacco cessation
Ontario	Environment, Heredity and Cancer	Factsheet	Cancer Care Ontario	Cancer risk factors
Ontario	Honouring the Métis path of well-being—breast screening	Factsheet	Cancer Care Ontario	Screening for breast cancer
Ontario	Honouring the Métis path of well-being—colorectal screening	Factsheet	Cancer Care Ontario	Screening for colorectal cancer
Ontario	Honouring the Métis path of well-being—cervical screening	Factsheet	Cancer Care Ontario	Screening for cervical cancer
Ontario	ON Cancer Treatment—Feel Good, Quit Smoking	Factsheet	Cancer Care Ontario	Quitting smoking during cancer treatment
Ontario	ON Métis Reduce Your Cancer Risks	Factsheet	Cancer Care Ontario	Prevention Behaviour risk factors
Ontario	Quit Smoking	Factsheet	Cancer Care Ontario	
Ontario	What is Cancer?	Factsheet	Cancer Care Ontario	General cancer information Behavioural risk factors
Ontario	Check-up for Prevention	Flashcard	Cancer Care Ontario	Prevention General cancer information
Ontario	Healthy Weight—Eat Right	Flashcard	Cancer Care Ontario	Prevention
Ontario	Stages of Cancer	Flashcard	Cancer Care Ontario	General cancer information
Ontario	Screening Q&A	Flashcard	Cancer Care Ontario	Screening general information
Ontario	Activity and Exercise	Flashcard	Cancer Care Ontario	Prevention
Ontario	Drinking Alcohol	Flashcard	Cancer Care Ontario	Prevention
Ontario	Cancer 101 Toolkit for FNIM People	Toolkit	Cancer Care Ontario	General cancer information Prevention screening
Ontario	Tools for the Journey: Palliative Care in First Nations, Inuit and Métis Communities	Toolkit	Cancer Care Ontario	Palliative care Grief
Ontario	Let’s take a stand against colorectal cancer!: Community Learning Series Reference Manual	Reference manual	Cancer Care Ontario	General cancer information Colorectal cancer Prevention screening
Ontario	Métis Cancer Survivor Story	Video	Cancer Care Ontario & Métis Nation Ontario	Métis survivor story
Ontario	Healing and Wellness—Cancer Care	Website	Métis Nation Ontario	General cancer information Breast screening Cervical screening

**Table 7:** Summary of programs and services for FNIM cancer patients in Canada.

Organization	Title	Service Purpose	Services Available
**Alberta**			
Alberta Health Services	Alberta Health Indigenous Wellness Core	Provide culturally appropriate cancer care to Indigenous cancer patients and families.	Nurse navigators, Indigenous-specific cancer resources, traditional wellness and cultural support.
**British Colombia**			
BC Cancer	Indigenous Cancer Control	Supporting Indigenous cancer patients, survivors and their families.	Liaison positions at BC cancer centers to navigate Indigenous patients and families; culturally appropriate screening information campaign.
**Manitoba**			
Cancer Care Manitoba	Manitoba Underserved Populations Program (part of the Community Oncology Program)	Help people who, due to geography, language, culture, or other barriers, may have trouble getting screened for cancer or receiving cancer treatment and support	Education and Liaison Nurse works with FNIM patients and families
**Ontario**			
Kingston Health Sciences Centre	Aboriginal Peoples with Cancer	Support Aboriginal people with cancer by someone of Indigenous ancestry	Patient navigator; All Nations Healing room.
Cancer Care Ontario, Southeast Regional Cancer Program	Aboriginal Navigator	Provide services to FNIM patients and family throughout the cancer journey from diagnosis, treatment, recovery to palliative in Southeast Ontario health region	Patient navigation during appointments, for healing options including traditional healing.
Mount Sinai Hospital, Toronto	Coping with Cancer/Indigenous Patient Navigator	Support Indigenous cancer patients throughout their cancer journey	Assistance with travel to treatment, connect Indigenous patient with spiritual and community support, assist with financial support, connect with care providers.
Ottawa Hospital	Indigenous Cancer Program	Improve the performance of the cancer system with and for First Nations, Inuit and Métis peoples in Ontario; Ensure delivery of culturally sensitive and safe care	Patient navigation for continuity of care, language translation, clinic visits, spiritual support, culturally safe care; Windocage Community Room.
Cancer Care Ontario and Toronto Central Regional Cancer Program	Indigenous Patient Navigation Specialist—Toronto Central Regional Cancer Program	Provide support and advocacy for FNIM and urban Indigenous patients and families	Coordinate access to cancer services; address cultural and spiritual needs; network with partners to ensure a culturally safe experience.
Ontario Health, Cancer Care Ontario	Indigenous Tobacco Program	Education and support to reduce tobacco use	Distinctions-based approach to tobacco cessation through partnership with FNIM communities and organizations. Focus on prevention, education and cessation.
Cancer Care Ontario	Indigenous Navigators	Provide support and advocacy for FNIM and urban Indigenous patients and families.	Available in ten health regions; coordinate access to cancer services; address cultural and spiritual needs; network with partners to ensure a culturally safe experience.

**Table 8:** Recommendations determined by records and review findings.

Recommendations for Researchers, Research Institutions, Data Custodians and Clinicians
Fund and support Métis-specific cancer research. Collaborate with Métis Nations, organizations, and communities to ensure cancer research is Peoples-specific and relevant to Métis priorities and needs. Prioritize research that focuses on qualitative in-depth information about Métis experiences with cancer. Incorporate sociodemographic variables into analyses to show how social determinants of health, including colonization and systemic racism, relate to behavioural risk factor rates, cancer screening participation, cancer incidence and cancer survival rates, to help inform more targeted intervention efforts. Be precise in reporting on Indigenous populations by specifying when data are tied to individual Indigenous groups and avoiding pan-Indigenous claims when data are predominantly from one group. Develop partnerships and knowledge-sharing models among Indigenous governments, researchers, and data custodians. Apply ethnocultural identifiers to cancer registries in a way that ensures cultural safety. Link health, cancer, and Métis registry datasets to gather longitudinal Métis-specific cancer data for cancer surveillance purposes. Establish a coordinated approach to track Métis cancer data at a national level to provide a more comprehensive picture of how cancer is impacting Métis citizens, families, and communities, and to provide valuable information to inform clinicians, interventions, and support services.

## Appendix A

Data extraction template used in Covidence.

Summary

Title

Record type (peer-reviewed, non-peer-reviewed, program/service, educationalmaterial, policy/plan)

Jurisdiction

Authors

Publication date

Purpose/aim

Service provided & Targeted audience (if relevant)

Cancer continuum focus area

Notes

Methods

Research type (Quantitative, qualitative, mixed)

Methods used

Study population (FNIM, Métis only, etc.)

Population sample (#)

Data source

Collaboration with Indigenous entity (Y/N)

Outcomes/Key content

Outcomes/Recommendations

Key components of material/service/program/strategy

Key findings relevant to scoping review objectives
